# Towards higher-dimensional structured light

**DOI:** 10.1038/s41377-022-00897-3

**Published:** 2022-07-05

**Authors:** Chao He, Yijie Shen, Andrew Forbes

**Affiliations:** 1 Department of Engineering Science, University of Oxford, Parks Road, Oxford, OX1 3PJ UK; 2 Optoelectronics Research Centre, University of Southampton, Southampton, SO17 1BJ UK; 3 School of Physics, University of the Witwatersrand, Private Bag 3, Johannesburg, 2050 South Africa

**Keywords:** Optical physics, Optical physics

## Abstract

Structured light refers to the arbitrarily tailoring of optical fields in all their degrees of freedom (DoFs), from spatial to temporal. Although orbital angular momentum (OAM) is perhaps the most topical example, and celebrating 30 years since its connection to the spatial structure of light, control over other DoFs is slowly gaining traction, promising access to higher-dimensional forms of structured light. Nevertheless, harnessing these new DoFs in quantum and classical states remains challenging, with the toolkit still in its infancy. In this perspective, we discuss methods, challenges, and opportunities for the creation, detection, and control of multiple DoFs for higher-dimensional structured light. We present a roadmap for future development trends, from fundamental research to applications, concentrating on the potential for larger-capacity, higher-security information processing and communication, and beyond.

## Abstract (web summary)

It is the first perspective or review that considers the dimensionality that may be reached with structured light, as well as the opportunities and challenges in pursuing this.

## Graphical abstract

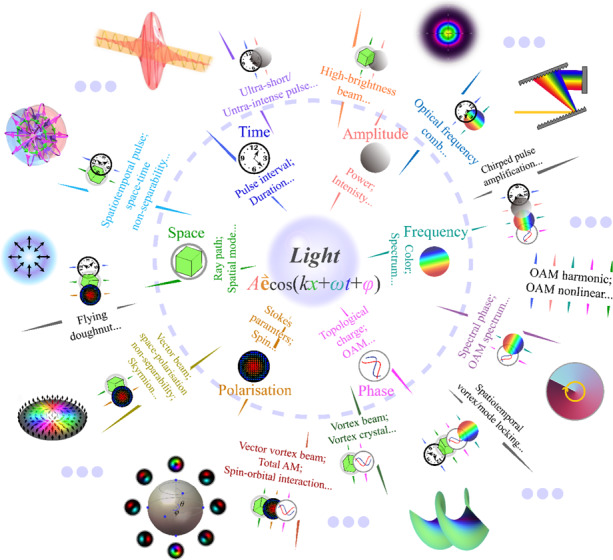

Our textbook description of light brings our attention to bear on its traditional form as an electromagnetic wave, comprising a wavelength and frequency, amplitude and phase, and with the direction of the disturbance (confined to the transverse plane) captured by its polarisation state. Yet light’s structure can be infinitely more complex, with many degrees of freedom (DoFs), each with a potential alphabet formed by its corresponding dimension. These forms of so-called structured light^1^, illustrated in Fig. 1, take us beyond the transverse plane for light tailored in 3D (all three electric field components), beyond space for 4D fields sculptured in space (3D), and time (1D), and beyond classical waves to quantum structured light.

**Fig. 1:**
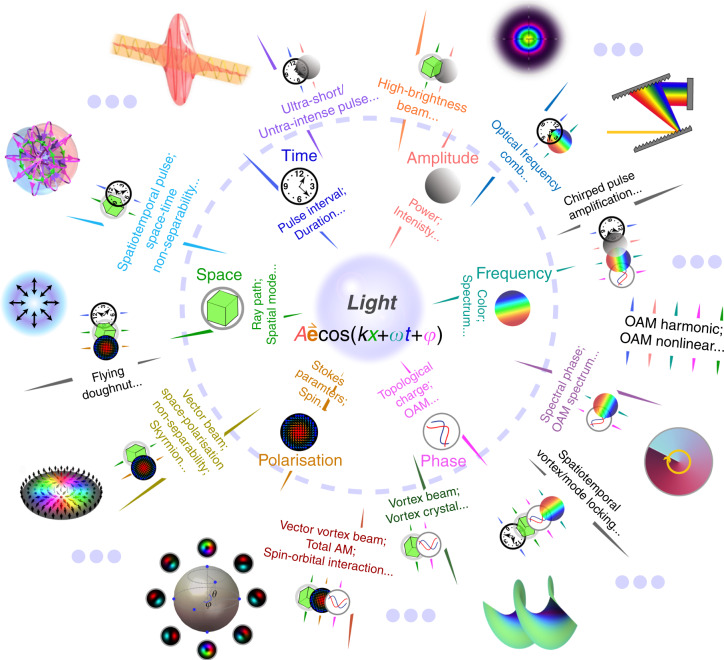
Light’s potential DoFs for control. By tailoring the on-demand structured light distributions of combined DoFs, various non-separable states can be produced so as to pioneer new research fields with theoretically unlimited dimensions to explore for structured light multiplexing

A topical example of this would be the evolution from polarisation states carrying spin-angular momentum (SAM) for a two-dimensional alphabet, to spatial modes that carry orbital angular momentum (OAM)^2^ for an infinite-dimensional alphabet^3,4^. That photons could carry OAM was known since the early days of atomic physics: while light intensity driven dipole electronic transitions (referred to as E1 transitions) are common and exchange one quanta of angular momentum in light-matter interaction, the necessary quadrupole transitions (referred to as E2 transitions) with 2 quanta of angular momentum, driven by gradients in the light’s intensity, were deemed too rare to be of practical relevance. Seminal work^2^ 30 years ago linking the OAM to the helical phase structure of light meant that OAM photons could be routinely created in common optical laboratories, a watershed moment for OAM and structured light alike. The fundamental nature of the DoFs likewise plays a role in how controllable they are. For example, OAM forms a discrete countable basis through the helical twisting of the wavefront, each twist giving rise to an extra quanta of OAM per photon, whereas the linear momentum of light is also infinite in dimension but in a continuous variable DoF. The true excitement in the field is in combining DoFs for exotically structured light. For example, SAM and OAM combinations have given rise to vector vortex beams, the natural modes of optical fibre, long known as textbook solutions and now realisable in the laboratory^5^. Concomitant with the creation is the need for detection and control. The challenge is to identify which DoFs can be controlled, to what extent, and with what toolkit.

Addressing this challenge, with the aim to exploit all of light’s DoFs, has seen the emergence of extreme structures of electromagnetic waves and a myriad of advanced applications^6–13^, such as optical tweezers and trapping, optical sensing and metrology, fast and secure optical communications enhanced imaging and microscopy, and advanced laser machining. In science too, structured light has allowed us to test and alter paradigms with the creation of non-diffracting, self-healing, and accelerating light fields and quantum-like classical light.

Fuelling further science and applications requires pushing the limits through structured light. But to exploit the potential requires some care. Adding complexity does not guarantee efficacy. For instance, scalar OAM modes are not the eigenmodes of conventional optical fibre, but vectorial combinations are; mode division multiplexing is a particular use of space, whereas space-division multiplexing is far more general. In these examples, the symmetry and capacity of the space the modes fill must be considered carefully in order to structure the light for the purpose. Outlining the advantages to doing so in the purpose of this perspective. To this end, we discuss methods, challenges, and opportunities for the creation, detection, and control of multiple DoFs for higher-dimensional structured light. We first outline a framework for understanding progress in structured light, and then review the toolbox, covering the present status and future needs. In particular, we consider methods of multiplexing light’s structure to realise higher-dimensional information transfer and storage. We point out present challenges and future opportunities, and offer a vision of what might be possible with photonic technologies that harness all of light’s properties.

## Higher-dimensional and multiple DoF classically structured light

**Fig. 2:**
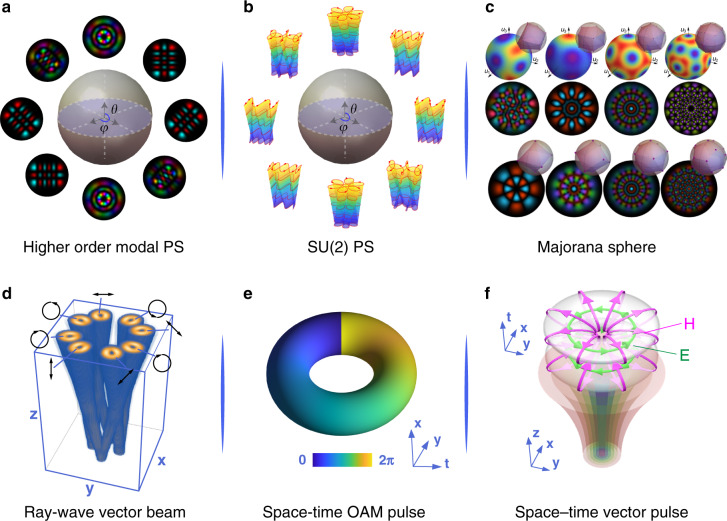
Structured light towards multiple DoFs and higher dimensions. **a** High-order PS representation of two-DoF 2D vector beams. **b** SU(2) PS representation of complex ray-wave geometric modes. **c** The Majorana sphere exploits hidden dimensions to extend complex structured Gaussian modes. Adapted from ref. ^18^. **d** Spatial vector beams with spatially dependent polarisation pattern. **e** Spatio-temporal vortex pulses with transverse OAM at space-time. **f** Flying electromagnetic toroidal pulses as space-time-polarisation non-separable states

Although the control of conventional structured light is limited to two DoFs—spatial mode and polarisation (Box 1), there exist ways to generalise the description of structured light to extend the DoFs (multidimensional) and dimensions (higher-dimensional). The conventional PS structure is an elegant tool to represent 2D state control of light. Note that the conventional structured light always means paraxial beams, while the tightly focused waves or evanescent waves may induce longitudinal components, which are expressed as 3D electromagnetic fields. However, there is still a number of spatial modes involved with complex optical transformation beyond the 2D qubit states, routinely employed in quantum optics. For instance, the family of higher-order Hermite-Laguerre-Gaussian modes (HLG) acts as an astigmatic transient state between HG and LG modes, which cannot simply be represented by a superposition of two eigenstates, but actually a spatial wave-packet with a set of eigenstates, with attempts to establish an elegant geometric model to represent such a case, i.e., the modal PS representation^14,15^ shown in Fig. 2a. Complex spatial patterns are surely not limited by the HLG mode, and a more general class of wave-packets takes the form of SU(2) coherent states, proposed to exploit more parameters to access multidimensional light shaping, for example, a class of exotic 3D geometric pattern coupled to Lissajous-trochoidal geometric curves were created^16,17^, see Fig. 2b, whereby prior HLG modes are just special cases of the new family. Recently, the modal Majorana sphere was proposed to represent general structured Gaussian modes. In contrast to the prior PS model which represents a light pattern by a specific point on the sphere, the Majorana sphere (Fig. 2c) depicts a structured mode by a set of points located on the sphere, revealing hidden symmetry to extend structured light^18,19^.

In addition to the route to find higher-dimensional representations of light modes, another meaningful approach is to explore multiple intrinsic DoFs, beyond the traditional spatial mode and polarisation, an example of which is the use of path as a DoF. In the context of structured light, this would be equivalent to using the linear momentum of light (its direction), easily created and controlled with just beam splitters. However, unlike the spin and orbital angular momentum just discussed, multiple linear momentum states imply multiple beams, forgoing the convenience of a single bright optical beam to control, so that resources scale with dimension. This serves to highlight that the challenge is not only more DoFs and dimensions but rather those that can be practically controlled. To deal with this, an effective tool of SU(2) symmetry was exploited to design ray-wave duality structured in paraxial beams^20^, where the wave patterns can be geometrically coupled to a set of caustic rays so as to open new DoFs to be controlled^21^ than prior vortex beams, for example, the number of rays, their directions and positions, and so on. We can also involve polarisation control into the ray-wave coupled states to access exotic ray-wave vector beams, see Fig. 2d, which enabled classical entanglement into multi-partite and higher-dimensional states^7^. So far the DoFs discussed are spatial, whereas time is also a DoF of light; combining the two allows for the creation of spatiotemporal structured light pulses. One such example is to combine OAM and “time” (Fig. 2e), for spatiotemporal vortices^22,23^. In contrast to the previous vortex beams where the OAM vector is along the propagation axis, the spatiotemporal vortex pulses can carry transverse OAM with a vortex in the space-time domain, promising new and anomalous spin-to-orbital physical effects to explore^24,25^. The further challenge is to structure light by simultaneously combining more DoFs, for example, a “flying doughnut” pulse as a recent state-of-the-art with a beautiful electromagnetic toroidal configuration in space-time (Fig. 2f), which was observed in an experiment very recently^26^. The toroidal pulses possessed a myriad of novel physics properties to explore, including space-time-polarisation non-separable states^13,27^, toroidal and anapole localized modes^28^, and complex topological and skyrmionic structures^29^. Therefore, it still requires designing new forms of structured light to realise light shaping in higher-dimensional space and more controllable DoFs to access new physical effects and advanced applications.

Box 1 Conventional 2D structured light In prevailing tutorials^104^, spatially structured light is regarded as a transverse electromagnetic wave and thus in the paraxial limit, the structured light beam can be described on the transverse plane (x, y) with a third dimension (z) for the propagation. Under this assumption, the most general case of a structured light field would be vector states of light, by assigning to each eigen polarisation component (Left- or Right-handed circular polarisations) a unique complex-valued field, $\left| \psi \right\rangle = \cos \theta \left| {u_R} \right\rangle \left| R \right\rangle + \sin \theta e^{i\phi }\left| {u_L} \right\rangle \left| L \right\rangle$, where *u*_*R*_ (*x*,*y*,*z*) and *u*_*L*_ (*x*,*y*,*z*) represent arbitrary beam modes fulfilling the paraxial wave equation, such as the Hermite-Gaussian (HG) and Laguerre-Gaussian (LG) modes. It is commonplace to use Dirac notation to express the field because the paraxial wave equation shares the same formation as the Schrödinger equation^233^. This expression accommodates structured light families in a two-dimensional Hilbert space. It is well-known that the polarisation state in a two-dimensional qubit space is mapped on the Poincaré sphere, $\left| \psi \right\rangle = \cos \theta \left| R \right\rangle + \sin \theta e^{i\phi }\left| L \right\rangle$, where the |*R*〉 and|*L*〉 eigenstates are corresponding to SAM of light with|*σ* = ±1〉, as shown in a. Similarly with two-dimensional spatial modes: for instance, the opposite helicity OAM states of a light beam are spanned by the basis vectors $\left\{ {\left| \ell \right\rangle ,\;\left| { - \ell } \right\rangle } \right\}$, sharing the same qubit formation as polarisation, which can be mapped on a Poincaré-like modal sphere^234^, $\left| \psi \right\rangle = \cos \theta \left| \ell \right\rangle + \sin \theta e^{i\phi }\left| { - \ell } \right\rangle$, as shown in b. This Poincaré sphere topology also plays an important role in quantum optics, because it represents a unit of quantum information (qubit). The vector beam state can be described as the tensor product of these two spaces, combining the two DoFs together for the new state $\left| {\psi _{{{\mathrm{v}}}}} \right\rangle = \left| {\psi _{{{\mathrm{o}}}}} \right\rangle \otimes \left| {\psi _{{{\mathrm{p}}}}} \right\rangle$ which is now spanned by four states |*ℓ*, *R*〉, |*ℓ*, *L*〉, |–*ℓ*, *R*〉, |–*ℓ*, *L*〉, courtesy of the tensor product that returns all orthogonal combinations. We show, in c, one example of the resulting higher-order Poincaré sphere^235^. Due to the two DoFs (spatial mode and polarisation), we have a classical 2D non-separable state, where the spatial mode cannot be factored out from the polarisation DoF (e.g., as the product of a single spatial mode and a single Jones vector), reminiscent to the formation of a bipartite entangled state in quantum mechanics^6^, with the classical DoFs mimicking the quantum particles. Importantly, this sphere now represents the total angular momentum of light, SAM, and OAM.
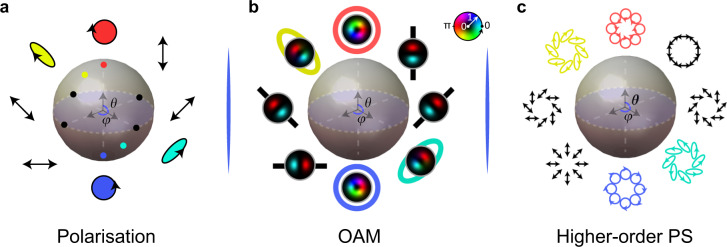

**The geometry of structured light**. **a** Poincaré sphere (PS) representation of the polarisation states of light. **b** The OAM PS of light. **c** The PS for higher-dimensional structured Gaussian modes.

### Higher-dimensional classical multiplexing

#### Dimensionality in optical-based information transport

**Fig. 3:**
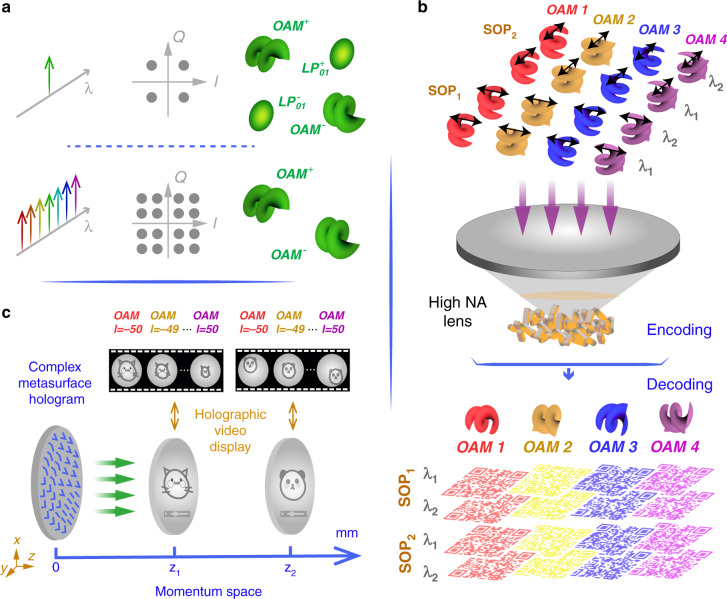
Illustrations of higher-dimensional structured light multiplexing. **a** Multiple wavelengths multiplexing in OAM-based optical communication. **b** Schematic of the OAM-based six-dimensional encoding and decoding process of nanometric QR codes utilising the combinations of four different OAM states. *P* polariser, *QWP* quarter-wave plate, *SOP* state of polarisation, *λ* wavelength. **c** Complex-amplitude OAM-multiplexing metasurface hologram with optically addressable holographic video display at two image planes

Optical communication has been an integral part of human society, from early communication by fire beacons to the first multi-level modulation during Napoleonic times. Over the past 200 years, “wire” based solutions have held supreme, from the early days of copper wire communications in 1812, through to optical fibre networks today. Optic communication solutions are rapidly reaching their capacity limit, requiring new degrees of freedom for packing information into light^30^. Here the many DoFs and dimensionality of structured light come to the fore. The idea is to exploit the spatial degree of freedom of light, referred to as space-division multiplexing (SDM)^31,32^ or its sister mode division multiplexing (MDM)^33^, for more channels and more capacity per channel, and has gained momentum in recent years (see refs., ^10–12,34–37^ for recent reviews). Topical among the multiplexing techniques is the use of OAM modes, particularly in conjunction with other DoFs^38^, as illustrated in Fig. 3a. Recently this DoF multiplexing has been extended to include path in novel ray-wave structured light to realise both ultrahigh capacity/speed and low bit-error-rate in communications^39^.

Free-space optical communication with structured light has enjoyed a resurgence of late because of its quadratic rather than exponential fall-off with distance, with the potential to bridge the digital divide in a manner that is license-free^40^. Developments followed advances in OAM, starting with a proof-of-principle demonstration down a corridor in the University of Glasgow^41^, with many seminal demonstrations quickly following including links without communication of up to 143 km^42^, and the use of 26 modes for petabits-per-second data rates^43^ and 80 gigabits per second over 260 metres^44^. However, there are still exited limitations for long-distance demonstrations, as the divergence and turbulence are maintaining challenges^45^. Later, the realisation that OAM is not necessarily ideal for free-space^45–48^ has seen the expansion from OAM to include the full radial and azimuthal LG basis^49,50^, Bessel beams^51,52^ and HG modes^53,54^, as well as vectorial light^55–57^.

Fibre-based communication has traditionally been restricted to single-mode fibre, hence only one pattern of light with a simple structure (scalar Gaussian modes). Conventional multi-mode (MM) and few-mode (FM) fibre can support many modes, even OAM, but at the expense of modal coupling^58^. In the first MDM, 10 m of MM fibre was used with the linearly polarised (LP) approximations to the true vectorial modes^33^ followed ~40 years later with all nine mode groups in a ~27 km fibre assisted by multiple-input-multiple-output processing^59^. The reach has since increased in these conventional fibres with three spatial modes over up to 6300 km^60^. Only recently have the tools been developed to customised fibre to modes^61^, opening the range of structured light possible. This has led to seminal advances including 1.6 Tbit/s OAM communication down custom ring core fibre^62^, 12 OAM modes over ~13 km of fibre^63^, eight OAM modes over 100 km^64^ and the expansion from scalar to vectorial modes in fibre^65^. In order to conquer the intensity loss within fibre—which is more significant than in free-space—amplification of structured light is required^66^, now reaching up to 18 across modest wavelength bands in fibre systems^67^.

#### Dimensionality in optical-based information storage

In addition to the multidimensional data transport, the DoF multiplexing is also revealed in optical-based information storage with improved speed/capacity and security. Historically, information recording and storage have experienced a technological evolution. The pathway has been undergone from paintings, carvings, scribing and digitisation, to optical compact discs (CDs), where an optical laser beam was used to store the binary data, which was also one important milestone in digital information technology^68^.

Such optical data storage methods (from CDs in 1980s, to nowadays digital video discs and Blu-ray discs) feature a limitation—the data is recorded and confined in a diffraction-limited region hence the capacity can be only reached into a few tens of gigabytes (GBs)^69^. The revolutionary developments of nanotechnology especially advanced nanophotonics, as well as exploiting multi-DoF multiplexing in structured light have been paving the new way for ultrahigh optical data storage capability beyond GBs.

Nanoparticles (such as gold nanorods) play an important role in modern multidimensional storage, featuring unique advantages such as polarisation selectivity and sharp spectral selectivity. They have continuously brought insightful multidimensional multiplexing possibilities for optical storages^70–73^. A remarkable breakthrough was made via harnessing three spatial dimensions, polarisation and wavelength to realise 5D light multiplexing^71^. Moreover, the demonstration was realised in a very compact volume with ultra-dense information density with an equivalent capacity of 1.6 TB in a single disc by exploiting the properties of longitudinal surface plasmon resonance of gold nanorods. This multiplexing data storage scheme was extended to 6D now, by exploiting OAM as an additional dimension^74^ (Fig. 3b). The technique essentially utilises synthetic helical dichroism and the polarisation aberrations of high numerical aperture lenses to enable OAM-dependent polarisation ellipses in a tightly focused beam, leading to explicit OAM sensitivity at the nanoscale for information storage. It highlights the exciting prospects in associating structured light with structured matter, for control at scales from the large to the small.

Metasurfaces-based nanophotonic platforms with multiplexing functionalities empower spatial light modulation for optical holography techniques, both linear^75–78^ and non-linear (see later section). The SAM-based multiplexing method obtained its perfection with the emergence of such technology, where two independent light fields are encoded onto a single metasurface and can be extracted by two orthogonal polarisations states^78^. Similar to the actions taken by various multiplexing techniques that moving point of view from SAM towards OAM, a wide range of OAM-dependent holographic images with different helical mode indices is proposed as a milestone^76^, opening a new window towards higher-dimensional structured light multiplexing. With the capability of multiplexing up to 200 independent OAM channels, a complex-amplitude OAM-multiplexing metasurface hologram (Fig. 3c) to achieve OAM-dependent orthogonal image frames with two holographic videos being simultaneously reconstructed is proposed as state of art^77^.

### Creation, control and detection

Optical cycles are much too fast to allow direct temporal light shaping, while direct modulation of the wavelength bandwidth lacks sophistication (mostly based on thin-film interference and absorption). For this reason, temporal light shaping reduces to spatial light shaping of the frequency components, which are usually path separated by a dispersive element (often a grating) before being recombined through the reciprocal process to construct the desired temporal pulse^79^. There are two salient points to infer from this example: (1) the importance of spatial creation and control of structured light, even for time-shaping, and (2) the importance of the reciprocity of light in the toolkit, which is often exploited for detection.

In the spatial domain, we may control the amplitude and phase of each polarisation component, the latter by propagation and geometric phase, both of which can be made polarisation specific. Conventional experimental techniques and devices for the generation and control of structured light include interferometric arrays, in which, various degrees of freedom are manipulated independently on each arm of the interferometer for a later on-axis recombination. In its most simple version, such approaches allow only to generate one single-mode at the time^80^. Early creation tools included polarisation independent propagation phase, either by refraction or diffraction. While refractive solutions have lost popularity of late, recent developments in free-form optics have given new impetuses to this direction, with unprecedented control possible^81^, even for miniature on-wafer elements through direct laser writing^82^. Even simple refractive elements can be tailored for vectorial light, as has been elegantly shown with glass cones^83^ and GRIN lens^84,85^, common elements in most optical laboratories and making clear that customised light does not always need customised tools.

In the early 1990s, there was an explosion of activity in diffractive optical elements (DOEs), to tailor light by interference and diffraction rather than by reflection and refraction, but these were mostly limited to scalar structured light. This has since been superseded by computer-generated holograms written to dynamic devices such as liquid crystal spatial light modulators (SLMs)^86,87^ and digital micro-mirror devices (DMDs)^88^, allowing both amplitude and phase control independently for each polarisation component. These rewritable solutions for the creation of on-demand vector modes with exotic polarisation and spatial distributions have propelled structured light studies worldwide.

Geometric phase has been exploited for complex spatially structured light^89^, by definition polarisation sensitive, allowing for the creation of scalar and vectorial light fields. Perhaps the most famous example is the use of so-called q-plates for control of conjugated-symmetry vector vortex beams^90^, which have found a myriad of applications^91^. Liquid crystal technology and its geometry has been extended significantly to include radial and azimuthal control through geometric phase-controlled amplitude tailoring^92^, and multi-spectral SLMs based on geometric phase^93^. A more recent move to subwavelength structures in the visible has allowed for polarisation-dependent propagation and geometric phase control using metasurfaces^94,95^, paving the way for all phases to be exploited. Key to this is the ability to create precise nanostructured matter to control and create structured light^96,97^. One example is the so-called J-plate for arbitrary spin-to-orbit conversion^98^ and the TAM-plate for arbitrary conversion in 3D^99^. Using OAM as an example, the state-of-the-art with this toolbox includes up to 200 simultaneous modes from a single device^100^, with mode number up to 600 using phase^101^ and 10,000 using amplitude^102^, and up to OAM of 100 in a vectorial mode^103^. Can these limits be pushed further? What is the impact on modal purity as modal number is increased? How can we reach the thousands of modes at high purity needed for optical communications? These questions remain open and challenging.

A promising avenue is to execute the creation step inside the laser cavity, rather than modulate the external field, with the benefits of enhanced purity, better efficiency, and compactness. The at-the-source solutions mirror the external shaping tools and evolution closely (see Ref. ^104^ for a review). Early work soon after the invention of the laser used amplitude filtering to differentiate the modes, for example, wires and apertures for HG and LG modes, respectively. Diffractive optical elements saw phase-only solutions for arbitrary complex scalar light, later extended to dynamic control with intra-cavity SLMs. It has been the desire for OAM modes from lasers that has fuelled modern laser developments^105,106^. Only recently have we seen the use of geometric phase and spin-to-orbit conversion for laser mode control^107^, and recently lasers based on metasurfaces^103,108^. On-chip devices have often had success with the geometry and topology of the micro-structure, producing robust topologically stable light sources^109^, compact OAM sources^110^, and spin controlled OAM lasers^111^. It is possible to exceed these numbers by exploiting degenerate cavities to produce coherent and incoherent sums of hundreds of thousands of spatial modes for complex forms of multimodal light directly from the source^112,113^. Here the exciting avenue is not only the laser as a source of complex structured light, but that the laser itself is a complex problem solver, where the answer lies in the very structure of the output light^114^.

Despite the impressive advances, most solutions can only tailor two-dimensional bipartite vector vortex states of light. While the recent TAM-plate technology has extended this to 3D, it is hard to control higher-dimensional states and go beyond the present two DoFs (spatial mode and polarisation). A recent approach to obtain high-dimensional structured light has been to extend the DoFs to include path, a DoF often exploited in quantum optics but not yet fully explored in the context of classical structured light. This has been done both external^115^ and internal^21^ to lasers. Combining internal and external control has seen the production and control of four DoFs in eight dimensional classically structured light^7^, for the classical equivalent to the quantum tripartite GHZ states. The ultimate holy grail of all techniques is the full control of the multiple DoFs of light into designed higher-dimensional state with high purity, which enables the on-demand generation of quantum analogue modes.

Detection of structured light is typically executed as the reciprocal of the creation process, by either a modal filter or a modal mapping. Modal filters can be simple distorting devices such as triangular apertures^116^ or tilted lenses^117^ (both used extensively for OAM), and easily extended to other mode families. The idea is to recognise the altered intensity map and infer the original, a process that can be improved further with machine learning approaches^118,119^. More sophisticated approaches exploit an optical inner product for a quantitative measure and reconstruction of any scalar or vectorial structured light field (see Ref. ^120^ for a recent tutorial). The so-called “match filter”, originating from the pattern recognition community of days gone by, is simply the conjugate of the creation phase, exploiting the reciprocity of the creation step: if a known beam X can be shaped into another known beam Y, then by reciprocity if Y is the incoming unknown beam then only this solution will map back to X and result in a detection. These approaches are filters since only one of the many incoming modes can be detected at a time with full signal, or the signal is split into multiple channels for reduced signal to noise^121^. Many compact filters based on dynamic and geometric phase have been implemented, and form the heart of many demultiplexing solutions in optical communication. Modal mappers on the other hand are in principle deterministic, conformally altering one mode into another. As such they can be viewed as lossless creators and lossless detectors of structured light. The solutions have to be found from first principles, and here the task is very challenging as no direct recipe exists for arbitrary structured light. Instead, particular solutions have been found for OAM modes^122^, Bessel modes^123^, radial LG modes^124^, general LG modes^125^, HG modes^126^, multipole phases^127^ and vectorial OAM modes^128^. Recent work has exploited this form of transformation for the control of structured light, including multiplication and division of OAM classical^129^ and high-dimensional quantum gates^130^, borrowing concepts from photonic lanterns in fibre optics. Presently, we have no deterministic universal mode converter for the creation and detection of structured light, a major stumbling block in applications where the light must be tailored on-demand.

### Higher-dimensional quantum structured light

**Fig. 4:**
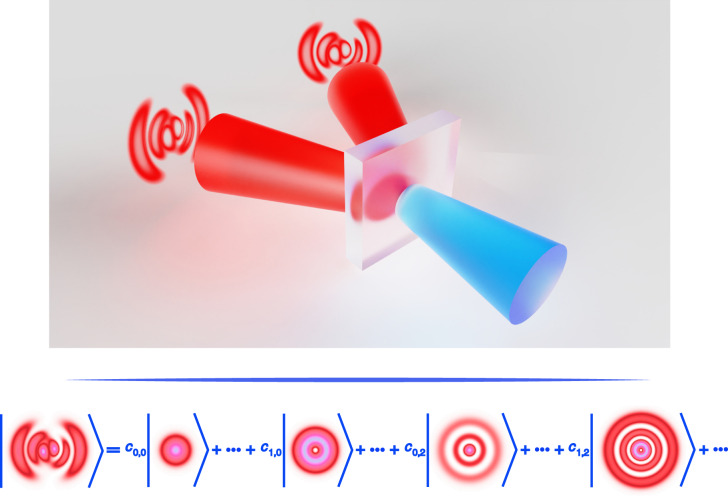
A typical quantum toolbox. The core quantum toolbox (top panel) typically exploits the generation of entangled photons, shown in red, by spontaneous parametric downconversion (SPDC) of a high-energy photon (shown in blue), often using non-linear crystals. Expressing and controlling the entanglement in the spatial basis comprising patterns of light (bottom panel) allows for high-dimensional entanglement to be exploited in quantum protocols. The more patterns in the superposition, the higher the dimensionality of the quantum state

In addition to the classical advances outlined, the quantum states of structured light have likewise seen tremendous developments and applications^131,132^. The workhorse in many quantum optics experiments is spontaneous parametric downconversion (SPDC), illustrated in Fig. 4 (main panel), where one high-energy photon shown in blue is downconverted to produce two lower energy entangled photons shown in red. The entanglement is ensured by the phase-matching conditions of the crystals, expressed naturally in the linear momentum basis. Since entanglement does not change with a change of basis, one can alter the basis to that of orthogonal structured light modes. This is illustrated in Fig. 4 (bottom panel) for one of the two photons: the photon is in a superposition of many spatials modes, each with some complex weighting. The number of such modes determines the dimensionality of the single photon state. The tensor product of the two photons’ states then returns the bi-photon entangled state. For example, in the OAM basis the final bi-photon state (of photons A and B) is written as |*ψ*〉_*AB*_ = | 0〉|0〉 + |1〉|−1〉 + |−1〉|1〉 + ···, with each single photon superposition as |*ψ*〉_*A*_ = | 0〉 + |1〉 + |−1〉 + ··· for photon A, and similarly for photon B. The dimensionality is determined by the choice of basis, the crystal parameters, and optical delivery system’s modal bandwidth (how many spatial modes can pass through it) and notably, the detection system.

Since the “creation” step in quantum is “detection”, it can be tailored to customised the desired quantum state by post-selection of a specific basis, resulting in entanglement of LG, HG and Bessel modes (see Ref. ^133^ for a review). Unfortunately most of the quantum detection toolkit is based on linear optical elements and filters, making the process probabilistic and thus negating the benefit of the high-dimensional space. For quantum light, where only one detection is possible per photon, the challenge is compounded by the time to accumulate statistic, often requiring many measurements to reconstruct the quantum state. Having post-selected a state, the real “detection” is to quantify what has been made. Quantum state tomography is the standard tool for 2D^134^ and high-dimensional spaces^135^, but scales unfavourably with dimension. Entanglement witnesses^136^ are faster but not quantitative, and many do not work in arbitrary dimensional spaces, or are basis dependent, fuelling the development of a modern toolkit that is fast and quantitative^137^, but with much work yet to be done.

The first quantum entanglement experiment with structured light exploited OAM in analogy to SAM for multiple qubit spaces^138^. Since then, structured light has been used to access high-dimensions using spatial modes for quantum key distribution, first with entangled states in five dimensions^139^ and later with single photons in seven dimensions^140^. Using spatial modes beyond just OAM has demonstrated four dimensional self-healing entanglement with Bessel beams^141^, engineering of high-dimensional spatial states by Hong-Ou-Mandel interference^142^, and high-dimensional Bell violations^143^. The state of the art includes 100-dimensional states in one DoF^144^, ten photons entangled in two DoFs^145^, three photons with OAM and hybrid states, entanglement swapping with qubits of OAM^146^, teleportation in three dimensions with path^147,148^ and ten dimensions with OAM^149^, and quantum secret sharing in eleven dimensions^150^. Following its classical counterpart, hybrid spin-orbit quantum states have become popular since their seminal introduction^151^, and have been used for quantum information processing and communication^152–154^. The present challenge is not in creating the desired dimensionality, but in transporting it intact across a channel, for example, for secure communication across free-space or optical fibre. In contrast to unstructured light, which has reached 4600 km in a combined free-space and fibre network^155^, structured quantum light languishes at distances in the order of 300 m in free-space^152^ and low kms in optical fibre^153^. The challenge is to find robust states of quantum and classical light for such channels, or efficient means for error correction, so that high-dimensional classical and quantum communication approaches the same reach as its unstructured counterpart.

### Challenges and opportunities

On the one hand, methods to further boost the multiplexing DoF are always of high demand. On the other hand, many practical factors such as the robustness of the technique, and the complexity and cost of the device, need to be taken into consideration. Here we discuss several challenges and provide potential solutions. Finally, we conclude this paper with future prospects for open discussion.

#### Possibilities for higher dimensionalities

We argue that there is much potential to further push the limit of structured light. This is based on the fact that several widely appreciated dimensionalities can possibly pave new ways for multiplexing. For example, time has seldom been exploited as an independent DoF for the above technique. Recent advances have highlighted certain new forms of spatiotemporal structured light, such as spatiotemporal vortex^22^, light pulses with strong spatial-temporal inseparability^27,156^ and even spatial-temporal-polarisational inseparability^26^, which indicates that the dimension of time may be adopted as another powerful DoF to benefit current techniques. Moreover, the introduction of ray-wave coupling in structured light represents that the optical modes can be described by both wave diffraction and geometric rays^21^. By applying ray-wave duality, wave patterns can be carried in sub-ray space in the paraxial regime, the light control of which is analogous to high-dimensional quantum states^7^. In turn, tools borrowed from quantum mechanics also exhibit great potential for digging out hidden DoFs of light^157^. In addition, light shaping beyond the linear regime towards non-linear interactions is a topical way to nurture higher-dimensional control^1^, and intriguing possibilities also exist in harnessing the state of polarisation located inside the Poincaré sphere (well-known as depolarised state^158^). These existing sectors show great potential to serve as extra DoFs for further multiplexing and well deserve to be explored further.

The recently emerged “ray-wave duality” of light was also a promising effect to extend the dimensionality^20^. The idea is that carefully crafted spatial mode can appear to be both wave-like and ray-like, connecting wave optics and geometric optics. In the wave picture, the beam is a coherent laser mode and so can be imbued with typical structured light features. On the other hand, the ray picture opens new DoFs to be controlled, for example, the amount of rays, their directions and positions, and so on, so as to extend the dimensionality that the pure wave optics does not have.

#### Information precision—various optical aberrations need to be conquered

**Fig. 5:**
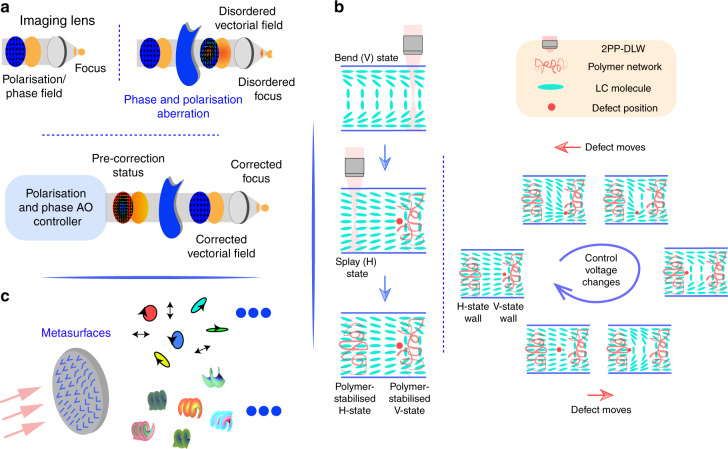
Advanced optical technique assisting structured light multiplexing. **a** A perfect optical system and an aberrated system, as well as vectorial-AO-enhanced optical system. **b** Electrically tunable disclination line—visualisation of the movement of different topological defect states under different control voltages. **c** Metasurface-based techniques for complex beam multiplexing

Commonplace optical components such as imaging/focusing lenses, beam splitters, and protected silver mirrors can contribute to vectorial (polarisation and phase) optical aberrations, in conjunction with other issues such as external turbulence^84,159–167^. The correction of aberrations is crucial for both light illumination and signal detection, as the induced phase and polarisation errors can cause detrimental degeneration of information such as image contrast, OAM phase distribution (purity) and correctness of vectorial information. They are vital for light multiplexing, such as direct OAM and/or polarisation multiplexing^74,168^. The novel adaptive optics (AO) technique for both phase and polarisation errors correction (Fig. 5a) have great potential to provide a solution for such problems. While AO techniques have been used for dynamic feedback correction of phase aberrations for various optical systems spanning from aerospace to microscopy^169,170^, e.g., OAM communication systems^171^, besides other alternative techniques such as electronic digital post-processing, the feedback correction strategy to incorporate polarisation control is newly-launched^164,172^. The advanced vectorial-AO technique is therefore at a position to further assist applications of structured light in conquering phase and polarisation distortions. Furthermore, we note that the concept of modes in AO techniques also features great potential to act as extra DoFs, considering its independently controllable property. The prospective spans across traditional phase and polarisation modes to full vectorial modes^170,173^.

#### The ability of extra-dimensional manipulation for the development of novel optical devices

As we mentioned before, higher-dimensional structured light multiplexing is recently adopted via central components such as optical fibres, metasurfaces, SLMs and DMDs. Although these devices have already been successfully used in various scenarios, new devices featuring extra modulation dimensions, robust performance, competitive mass, and size, or precise dynamic modulation ranges, have always been in high demand. The advanced liquid crystal (LC) devices enabled by direct laser writing (DLW) technique should be a possibility. DLW, which is a powerful non-linear fabrication technique, has been adopted to generate novel 3D, reconfigurable LC templates for cost-effective, high flexibility structured light field generators^174,175^. Recently [Fig. 5 (b)], in-situ DLW enables polymer structures to be fabricated directly inside electrically addressable LC devices to lock in voltage-dependent topologically discontinuous states^176^. These discontinuous states, which are generated using devices with topological pixels, potentially provide possibilities for novel multiplexing DoFs. The advanced techniques based on metasurfaces for the creation of structured light also have attracted public attentions when conducting beam multiplexing^75^ (Fig. 5c), taking advantage of their capabilities such as high efficiency.

#### New forms of light

**Fig. 6:**
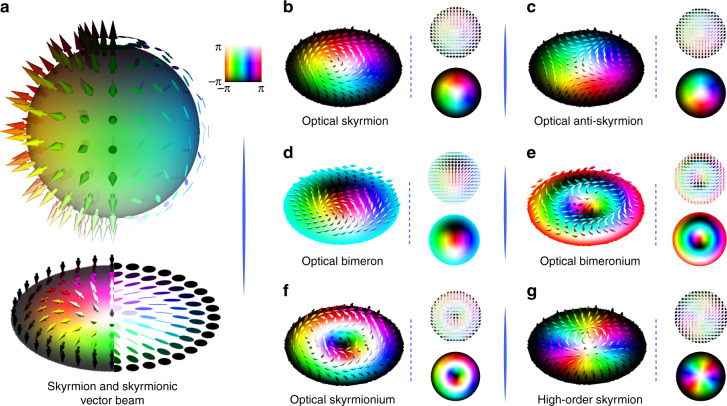
Optical Skyrmions. **a** PS and Bloch sphere, Skyrmion and Skyrmionic vector beam. **b**–**g** are several representative optical skymionic fields (Skymion, anti-Skymion, Bimeron, Bimeronium, Skyrmionium, and high-order Skyrmion); the corresponding Stokes vector fields are also illustrated. The hue colour means the degree of azimuth of polarisation ellipse, and the darkness and brightness mean the ellipticity from left-hand circular polarisation to right-hand circular polarisation

It is also highly topical to explore higher-dimensional structured light by referring models from different disciplines, e.g., topology, particle physics, and condensed matter. Here we would like to point out a newly emerging direction related with optical skyrmion—which may push the multiplexing limit further. Skyrmions are a kind of quasiparticle carrying a topological spin texture that originate from particle physics and magnetic materials^177^, with sophisticated hedgehog-like textures (see configurations in Fig. 6), and have recently been used as a powerful tool to tailor multidimensional structured light. Geometrically, a skyrmion can be simply understood as a topologically stable 3D vector field confined within a local space^177^. The main challenge in constructing an optical skyrmion is to find 3D vector components in non-transverse optical fields, which can be overcome by different approaches, e.g.,

1. Plasmonic skyrmions: The first method to construct the vector texture is using the electric field of evanescent waves on surface plasmon polaritons (SPP). By sculpturing structured gratings as a confined region on a metal film, the SPP field can form geometric standing wave fulflling the skyrmionic structure^178,179^. In addition to the electric field, the optical spin-angular-momentum fields in the SPP field was also proved to have the ability to construct skyrmionic texture^180,181^. It is emerging direction to design more general higher-order types of skyrmions with robust geometric and topological control^182,183^.
2. Free-space skyrmions: Conventional continue-wave beams are treated as pure transverse waves, where electromagnetic vectors are always 2D in-plane, which cannot be used to construct skyrmion. Recently, some new forms of optical modes possessing 3D vector fields were solved, which can be exploited to tailor skyrmionic textures, such as the 3D electromagnetic vectors to construct skyrmions in supertoroidal structured pulses^29,184^, the 3D optical spin-angular-momentum fields in tightly focused structured waves^29,184^, as well as the 3D Stokes vectors of vector beams^185–188^. Figure 6 shows the diversified Stokes-vector skyrmions constructed by complex vector beams, where each polarisation state corresponds to a Stokes vector of a certain 3D azimuth (based on Poincare sphere), and the polarisation pattern can be tailored to fulfil diverse skyrmion textures. The topologically protected property and robustness of the Stokes skyrmions have been demonstrated^185,186,188^.

More recently, it is a hot topic to find optical skyrmions with more kinds of topological textures using new kinds of optical vectorial fields. Such particle-like topological light fields have promised additional and extendable topological control for advanced applications, broadening the frontier of modern fundamental and applied physics. The most fascinating potentials of optical skyrmion configuration include (1) its ultra-small deep-subwavelength structure to upgrade the super-resolution imaging and microscopy, and importantly, (2) its wide range-tunable and diversified topology which features the great potential for higher-dimensional topological state control of light, and have great potential to provide new insight for further breaking limits of optical encoding, multiplexing, communication and encryption.

#### Towards a non-linear toolkit

Non-linear optics for the creation of classical structured photons has a long history, dating back to seminal work on OAM 25 years ago^189^. But it has only been recently that the field has taken off, as linear optical solutions reach their limit. First following traditional conversion of structure from one wavelength to another^190,191^, the toolkit quickly developed into structured light modal control. Today full control of light’s DoFs via multiple non-linear processes is possible.

Traditionally the focus in non-linear optics has been on wavelength conversion, with the low efficiencies relegating the question of the light to only “how much” and not “what does it look like”. The introduction of spatial structure has opened a myriad of possibilities, and a new take on non-linear processes. For instance, it is possible to have a second harmonic generation (SHG) that is composed of the *product* of two different spatial modes, rather than the sum as we see in linear optics, for new exotic forms of structured light^192,193^. The path degree of freedom can also be used by mixing structure with direction inside the crystal^194,195^. Interestingly, the coupling is not only between light and matter, but between differences in structure of the fields themselves, particularly within a given family. For instance, the “untwisting” of the azimuthal phase of an OAM LG mode, in turn, alters the radial index^196^, with the rules governing this interaction only recently unveiled^197^, and shown to be true for wave mixing processes of any order^198^. Similar processes have been observed with HG modes^199^, Ince-Gaussian^200,201^ and Bessel-Gaussian modes^202^, confirming OAM conservation^87^ and exploring the selection rules of these families.

In the vectorial regime, frequency conversion of vector structured beams has been characterised as producing non-trivial scalar outputs^203,204^ as well as vectorial outputs that differ from the input^205^. In this sense, the inhomogeneous state of polarisation has been proposed as a control parameter for non-linear process^206^. Recently it has been shown that one can convert a vector beam in frequency while retaining the polarisation structure, but changing wavelength^207–209^, for faithful vectorial wavelength control.

A recent development is the use of structured matter for non-linear control of structured light. This includes phase-matching for multiple wavelengths by 3D periodical polling in photonic crystals^210^ and non-linear metasurfaces that combine wavelength conversion with wavefront control^211^ and non-linear metalensing^212^. Spin-orbit interactions in metasurfaces^213^ and conventional crystals^214^ likewise has been cast in a new light through the prism of non-linear interactions.

These exciting advances have fuelled new applications, including non-linear holography^215–217^, image encoding^218^, optical memories^219^, imaging^220^ and microscopy^221^, to name but a few.

Quantum structured light suffers from extremely low count rates when multiple photons are involved^145^, while the toolkit for the analysis of high-dimensional states is very much in its infancy^136,137^. The use of more DoFs has the potential to open up new approaches^222^, but needs much more work to be realised practically. The issue of robustness to noise is still a topic under intense research^223^, while storage of quantum information in the form of spatial modes is only just beginning to emerge^224^. A severe limitation is that most quantum experiments with structured light are based on post-selection of the state, whereas true quantum state engineering in arbitrary dimensions has not yet been demonstrated, but may come closer, by exploiting the path for structured modes^225^. A ubiquitous tool in transferring entanglement and engineering quantum states is the beam splitter, used to establish entanglement between independent photons, the heart of a quantum repeater. While ideal for 2D quantum states, this linear optical solution results in significant losses and entanglement degradation when the state is high-dimensional. For example, without ancillary photons, entanglement swapping of a high-dimensional state as shown in Fig. 4 would result in a mixed state (rather than a pure state) and reduced contrast in imaging^226^. The solution is to increase the number of SPDC sources, but this route has little prospect for long-term success due to the very low efficiency of SPDC. Alternatively, an exciting and emerging approach is to use non-linear optics for high-dimensional state creation, control and detection. To this end, recent progress in classical pump shaping to control entanglement is gaining versatility^227^, and may be a simple future resource. This approach is based on non-linear optics at the source of the entanglement.

Non-linear detection schemes that replace our conventional linear solutions hold tremendous promise, and have the making of on-demand detectors for arbitrary classically structured light. The idea here is to exploit the structure of the “known” input beam and the “known” converted beam, to infer the structure of the “unknown beam. This has been shown using upconversion to detection structured modes without the need for a basis-specific detector^228^, and used for image enhancement^229^. Not only is the non-linear detector rewritable through the pump beam, but it also allows the detected mode to be transferred to a more convenient wavelength window, e.g., up-converting infra-red light so that it is detected in the visible. In the quantum realm, such approaches have already shown that they can overcome the ancillary photon limitation in quantum teleportation and entanglement swapping^149^ and extend new dimension control^230^.

Non-linear optics may also solve a pressing issue in classically structured light: all the aforementioned solutions are at low power levels. A promising prospect to amplify the low power states, which has already seen developments in thin disk, fibres, and bulk crystals, with recent state-of-the-art mimicking amplification of ultrafast lasers to demonstrate vectorial light parametric amplification in a polarisation insensitive manner, reaching 1000-fold amplification factors^231^. The convergence of structured artificial matter in the form of metamaterials with non-linear response^232^ with structured light creation, control, and detection, will surely fuel-efficient and compact solutions for high-dimensional classical and quantum states of light.

## Closing remarks

The explosive developments in structured light can be traced back to the seminal work^2^ in 1992, now celebrating 30 years of progress. Rather than slowing down, we are experiencing a renaissance in structured light, enabled by novel concepts on the nature of light itself that takes us beyond OAM, fuelled by a cutting-edge toolkit for classical and quantum states alike. Although the combination of DoFs and dimensions requires much further work, the future is surely a transition from the laboratory to new practical applications based on our new-found controllable DoFs and dimensions, promising impact from science to application.
